# Awareness, perspectives and practices of antibiotics deprescribing among physicians in Jordan: a cross-sectional study

**DOI:** 10.1080/20523211.2024.2378484

**Published:** 2024-07-19

**Authors:** Rana Abu-Farha, Lobna Gharaibeh, Karem H. Alzoubi, Rawand Nazal, Mohammed Zawiah, Ammena Y. Binsaleh, Sireen Abdul Rahim Shilbayeh

**Affiliations:** a Clinical Pharmacy and Therapeutics Department, Faculty of Pharmacy, Applied Science Private University, Amman, Jordan; b Biopharmaceutics and Clinical Pharmacy Department, Faculty of Pharmacy, Al-Ahliyya Amman University, Amman, Jordan; c Department of Pharmacy Practice and Pharmacotherapeutics, University of Sharjah, Sharjah, UAE; d Department of Clinical Pharmacy, Faculty of Pharmacy, Jordan University of Science and Technology, Irbid, Jordan; e Department of Clinical Practice, College of Pharmacy, Northern Border University, Rafha, Saudi Arabia; f Department of Pharmacy Practice, College of Clinical Pharmacy, Hodeidah University, Al Hodeida, Yemen; g Department of Pharmacy Practice, College of Pharmacy, Princess Nourah bint Abdulrahman University, Riyadh, Saudi Arabia

**Keywords:** Antibiotics, deprescribing, knowledge, barriers, attitude, Jordan

## Abstract

**Background:**

Antibiotics have significantly reduced mortality and improved outcomes across various medical fields; however, the rise of antibiotic resistance poses a major challenge, causing millions of deaths annually. Deprescribing, a process that involves discontinuing unnecessary antibiotics, is crucial for combating this threat. This study was designed to assess the knowledge, perceptions, and practices of physicians regarding antibiotic deprescribing in Jordan.

**Methods:**

A cross-sectional survey was conducted between January-February 2024 to assess the knowledge, perceptions, and practices of physicians regarding antibiotic deprescribing in Jordan. An electronic questionnaire served as the data collection tool. Descriptive analysis was performed using SPSS software version 26. Additionally, logistic regression analysis was carried out to identify independent factors associated with physicians’ willingness to deprescribe antibiotics.

**Results:**

The study involved 252 physicians, primarily male (n = 168, 67.7%), with a median age of 33 years. Regarding antibiotics deprescribing, 21.8% (n = 55) expressed willingness to deprescribe inappropriate antibiotics.

High awareness of deprescribing was evident, with 92.9% (n = 234) familiar with the concept, 94% (n = 237) knowledgeable about appropriate situations, and 96.8% (n = 244) recognising its potential benefits. Furthermore, 81.8% (n = 205) reported having received formal training in antibiotics deprescribing, and 85.3% (n = 215) were informed about the availability of deprescribing tools.

Physicians highlighted challenges including insufficient time (44.4%, n = 112) and resistance from patients (41.3%, n = 104) and colleagues (42.1%, n = 106). Despite challenges, a significant proportion regularly assessed antibiotic necessity (46.9%, n = 117) and educated patients about antibiotic-related harms (40.5%, n = 102). Logistic regression analysis revealed no significant demographic factors influencing physicians’ willingness to deprescribe antibiotics (*p* > 0.05).

**Conclusion:**

Physicians in Jordan exhibit high awareness of antibiotics deprescribing and recognise its benefits. Challenges such as time constraints and communication barriers need to be addressed to facilitate effective deprescribing practices. Comprehensive guidelines and interdisciplinary collaboration are essential for promoting judicious antibiotic use and combating antimicrobial resistance.

## 1. Introduction

Antibiotics inauguration is deemed a major medical revolution and one of the main modern medicine approaches to fight infections, lowering death rates, and addressing various infectious disease-related problems (Gould, 2016; Mohr, 2016). Antibiotics have played a crucial role in the success of numerous medical fields, including rheumatology, cancer, and invasive operations such as organ transplantation (Ventola, 2015). The accessibility of antibiotic therapy has greatly decreased mortality, raising average life expectancy (Adedeji, 2016).

The 1930s to the 1960s were considered the ‘great era’ of antibiotics, during which numerous antibiotics were developed. Unfortunately, this era ended because researchers were unable to maintain the pace of antibiotic discovery in the face of emerging resistant pathogens (Nathan & Cars, 2014). A growing number of bacteria are developing resistance to several antibiotics already in use, leading to the emergence of multidrug-resistant bacteria (Tanwar et al., 2014). Antibiotic-resistant bacteria pose a serious challenge to current healthcare due to the lack of new antimicrobial medications and the rising number of treatment failures caused by multidrug-resistant bacteria (Read & Woods, 2014; Spellberg et al., 2013).

Antibiotic-resistant bacteria may arise from various causes, such as the overuse of antibiotics in humans and animals, over-the-counter antibiotic purchases, increased worldwide travel, inadequate hygiene, and antibiotic overprescribing (Llor & Bjerrum, 2014; Van Boeckel et al., 2015). In 2019, antibiotic-resistant bacterial infections were estimated to cause 1.27 million deaths globally and by 2025, approximately 10 million deaths are expected to occur annually (Aslam et al., 2018; O'Neill, 2016). Specifically, these types of infection have been shown to result in longer hospitalisations, increased mortality, and higher medical expenses (Conlon-Bingham et al., 2019; Cosgrove, 2006).

Antimicrobial prescribing is a complex process influenced by various elements, including the healthcare system, physicians, other healthcare professionals, and patients (Rodrigues et al., 2013). Strong evidence links the consumption of antibiotics to the presence of antibiotic-resistant bacteria in both the community and the individual (Costelloe et al., 2010). There is strong evidence that reducing cumulative antibiotic consumption lowers the likelihood of antibiotic-resistant bacteria (Llewelyn et al., 2017).

When it comes to the management of infectious diseases, inappropriate antibiotic prescriptions include those written for unnecessary purposes, chosen incorrectly, or administered for the wrong duration of time (Alsayed et al., 2022; Fu et al., 2023; Mahmood et al., 2022). In such cases, deprescribing may be considered for the patient's benefit to prevent adverse reactions from recurring. Deprescribing is defined as the process of discontinuing an unsuitable medicine under the supervision of a medical professional to manage polypharmacy and enhance results (Woodford & Fisher, 2019).

Reports indicate that antibiotic-resistant bacteria are widely present in Jordanian hospitals. Methicillin-resistant Staphylococcus aureus (MRSA) is particularly common, with a rate of 45% (Karasneh et al., 2021). Various strategies have been developed to decrease the improper use of antibiotics in healthcare settings. In Jordan, the majority of efforts have concentrated on decreasing the initiation of unnecessary antibiotic treatments and have targeted prescribers (Al-Taani, Al-Azzam, Karasneh, Ababneh, et al., 2022; Al-Taani, Al-Azzam, Karasneh, Sadeq, et al., 2022; Karasneh et al., 2021). However, no efforts have been made to stop the already-initiated inappropriate antibiotics.

In Jordan, deprescribing is primarily the responsibility of physicians. Therefore, to design guidelines and methods to optimise appropriate medication usage, it is crucial to investigate physicians’ knowledge and desire to support effective deprescribing. Hence, the purpose of this was to assess the knowledge, perceptions, and practices of physicians regarding the deprescribing of antibiotics in Jordan.

## 2. Methods

### 2.1. Study design, sample, and setting

This research is a cross-sectional study aimed to assess the knowledge, perceptions, and practices of physicians regarding the deprescribing of antibiotics in Jordan. The study was conducted between January-February 2024, among physicians in Jordan. The study population comprised physicians registered with the Jordanian Medical Association, encompassing a wide range of sectors including private practices, public hospitals, and clinics. Inclusion criteria allowed for any actively practicing physician registered in Jordan, irrespective of specialty or sector. There were no specific exclusion criteria, except that participants needed to be currently practicing physicians. The survey reached physicians across all major cities and regions in Jordan, ensuring comprehensive national representation.

### 2.2. Data collection

To mitigate selection bias and enhance the study's validity, we implemented several strategies during the data collection phase. Physicians were selected using a mixed approach combining convenience and snowball sampling to capture a diverse demographic and professional spread, which helps in reducing the impact of selection bias typical to convenience sampling alone. The survey was distributed through social media platforms such as Facebook, WhatsApp, and LinkedIn. Research assistants and medical school professors were also involved to extend the reach. The questionnaire included a brief introductory paragraph specifying the target group and ensuring that only eligible physicians participated.

### 2.3. Survey development

We designed a survey to comprehensively assess the knowledge, perceptions, and practices of physicians regarding the deprescribing of antibiotics in Jordan. The questionnaire underwent validation for content and face validity by two experts in clinical pharmacy. It comprised five sections. The first covers demographic characteristics (e.g. age, gender, specialisation, years of experience, country of graduation, and number of daily consultation), The second section comprises closed-ended questions assessing physicians’ knowledge of Antibiotics deprescribing. The third section includes questions exploring physicians’ perception towards antibiotics deprescribing, and the fourth section evaluates physicians’ perception towards the potential challenges against antibiotic deprescribing. The last section assesses physicians’ previous practice in antibiotic deprescribing.

A pilot study was carried out with five physicians to evaluate the questionnaire's format, clarity, length, and general impression. Following their feedback, modifications were made to some of the questions. The data from this pilot study were excluded from the final analysis. The internal consistency of the questionnaire was confirmed with Cronbach's alpha values of 0.722, 0.953, and 0.845 for sections three, four, and five, respectively, indicating satisfactory reliability of the items in these sections (Taber, 2018).

### 2.4. Sample size determination

The sample size was calculated based on the number of subjects per predictor needed for regression analysis of factors affecting physicians’ willingness to deprescribe antibiotics (Tabachnick & Fidell, 2006). Adhering to Tabachnick and Fidell guidelines for sample size calculation while conducting regression analysis, 5–20 subjects are required per each predictor (Tabachnick & Fidell, 2006), and since six predictors are hypothetically assumed to influence physicians’ responses in the current study, the minimum sample size required to ensure satisfactory statistical power was found to be equal to 120 participants.

### 2.5. Ethical considerations

Ethical approval was obtained from the Institutional Review Board (IRB) at the Applied Science Private University [Approval Number 2024-PHA-1], and electronic informed consent was secured from each participating physician. The study strictly adhered to the ethical standards outlined in the Declaration of Helsinki.

### 2.6. Statistical analysis

Data were compiled and analysed using the statistical package for social science (SPSS) version 26 (SPSS Inc., Chicago, IL, USA). Descriptive statistics were employed to summarise the distribution of variables. Qualitative variables were analysed using frequency and percentage, while continuous variables were analysed using median and interquartile range (IQR). Normality was checked using the Kolmogorov – Smirnov test and histograms, with *p* ≤ 0.05 indicating non-normal distribution.

Logistic regression analysis was carried out to screen for independent factors associated with physicians’ willingness to deprescribe antibiotics. Following simple logistic regression, any variable with a *p*-value < 0.250 was considered eligible for entry in multiple logistic regression analysis. Before conducting multiple logistic regression analysis, variables were checked for the absence of multicollinearity (i.e. Pearson correlation coefficient <0.9 for any two variables). A *P*-value of ≤0.05 was considered statistically significant. Regarding the internal consistency of the study survey, a Cronbach alpha value > 0.7 was considered to indicate an acceptable internal consistency (Taber, 2018).

## 3. Results

The study comprised 252 physicians, with a median age of 33 years and an interquartile range of 11 years. Of the respondents, 67.7% were male (n = 168). The majority held the title of general practitioner with varying years of professional experience (median = 6 years). A significant proportion (n = 183, 72.6%) graduated from Jordanian medical schools. Physicians reported various daily consultation numbers, with 46% (n = 116) seeing over 20 patients per day. Regarding antibiotics deprescribing, 21.8% (n = 55) were willing to deprescribe any inappropriate antibiotic during their practice (Table 1).

**Table 1.** Sociodemographic characteristics of the study sample (n = 252).

Parameter	Median (IQR)	Frequency (%)
Age	33.0 (11.0)	
Gender		
Male		168 (7.7)
Female		84 (33.3)
Title		
General Practitioner		141 (6.0)
Junior Resident (1st or 2nd year of residency)		33 (13.1)
Senior Resident (3rd or 4th year of residency)		38 (15.1)
Consultant		40 (15.9)
Years of experience	6.0 (9.0)	
Country of graduation		
Jordan		183 (2.6)
Others		69 (27.4)
Number of daily consultation		
<10		38 (15.5)
10–20		97 (38.5)
>20		116 (46.0)
Are you willing to deprescribe any inappropriate antibiotic during your consultation?		
Yes		55 (21.8)
No		174 (9.0)
I don’t know		23 (9.1)

Note: IQR: Interquartile range.

The study showed that most physicians in the sample exhibit substantial awareness of antibiotics deprescribing. Specifically, 92.9% (n = 234) were familiar with the concept, 94% (n = 237) were knowledgeable about appropriate situations for deprescribing, 96.8% (n = 244) recognised its potential benefits, and 91.7% (n = 231) were aware of potential risks. Moreover, 81.8% of the participants (n = 205) had received formal training in antibiotics deprescribing, and 85.3% (n = 215) were informed about the availability of deprescribing tools (Table 2).

**Table 2.** Physicians’ awareness about antibiotic deprescribing (n = 252).

Questions	Frequency (%)	
	Yes	No
Are you familiar with the concept of ‘antibiotic deprescribing’?	234 (92.9)	18 (7.1)
Are you aware about appropriate situations for antibiotic deprescribing?	237 (94.0)	15 (6.0)
Are aware of the potential benefits of antibiotic deprescribing?	244 (96.8)	8 (3.2)
Are you aware of the potential risks of antibiotic deprescribing?	231 (91.7)	21 (8.3)
Have you ever received a training on antibiotic deprescribing?	205 (81.8)	47 (18.7)
Are you aware about the availability of deprescribing tools?	215 (85.3)	37 (14.7)

Physicians’ perceptions regarding antibiotics deprescribing were investigated, revealing significant insights (Table 3). The majority (n = 229, 90.9%) recognised the importance of antibiotic prescribing in compacting antibiotic resistance, and a significant proportion (n = 216, 85.7%) believed it to be a feasible practice in their clinical setting. Notably, a considerable number (n = 200, 79.4%) acknowledged the influence of patients’ expectations, often leading to antibiotics overprescribing. Physicians thought that patients are generally open to antibiotic deprescribing recommendations (n = 115, 45.6%), and they believed that deprescribing has as potential to improve patient outcomes (n = 137, 54.4%), and felt confidence in communicating about deprescribing (n = 157, 62.3%). Concerns were expressed about potential complications and admission resulting from antibiotics deprescribing (68.3% agreed/strongly agreed). Furthermore, the majority of physicians (n = 206, 81.7%) believed that antibiotic deprescribing should be included in clinical guidelines, while 73.4% (n = 185) acknowledged that withdrawing antibiotics prescribed by colleagues is unethical.

**Table 3.** Physicians’ perception towards antibiotic deprescribing (n = 252).

Statement	Strongly agreed/Agreed (%)	Neutral (%)	Strongly disagreed/Disagreed (%)
Antibiotic deprescribing is an important strategy to combat antibiotic resistance.	229 (90.9)	14 (5.6)	9 (3.6)
Antibiotic deprescribing is feasible in my clinical practice.	216 (85.7)	30 (11.9)	6 (2.4)
There is pressure to overprescribe antibiotics due to patient expectations.	200 (79.4)	42 (16.7)	10 (4.0)
I believe that patients are generally open to antibiotic deprescribing recommendations.	115 (45.6)	73 (29.0)	64 (25.4)
I believe that antibiotic deprescribing may lead to improved patient outcome.	137 (54.4)	88 (34.9)	27 (10.7)
I feel confident in my ability to communicate with patients about antibiotic deprescribing.	157 (62.3)	85 (33.7)	10 (4.0)
I think that antibiotic deprescribing may generate an increase in complications and hospital admissions.	172 (68.3)	53 (21.0)	27 (10.7)
I think that antibiotic deprescribing should be included in clinical guidelines.	206 (81.7)	37 (14.7)	9 (3.6)
I believe that withdrawing the antibiotic course prescribed by another colleague is unethical.	185 (73.4)	50 (19.8)	17 (6.8)

**Figure 1.**
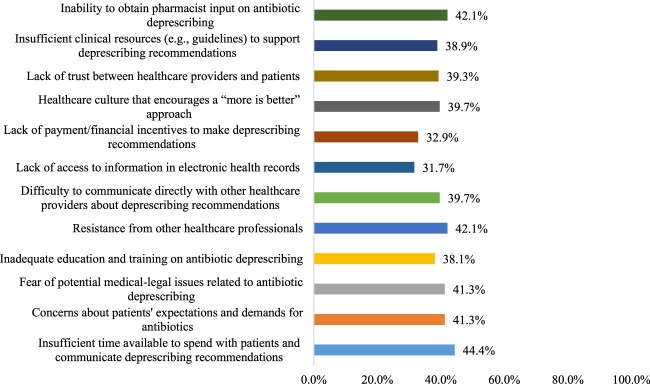
Potential challenges against antibiotic deprescribing as perceived by the physicians (n = 252).

Physicians perceived several challenges to antibiotic deprescribing, the most prominent being the insufficient time available to discuss deprescribing with patients (n = 112, 44.4%). Equally notable were barriers such as the inability to obtain pharmacist input and resistance from other healthcare professionals (n = 106, 42.1% for both). Concerns about medical-legal implications and patients’ expectations both present considerable challenges, each cited by 41.3% of physicians (n = 104). Other issues included disruption between providers and patients, a culture of more-is-better in healthcare, and communication barriers, each just under 40%. Less cited challenges involved the absence of financial motivation and restricted access to patient information in electronic health records, with proportions of 32.9% (n = 83) and 31.7% (n = 80), respectively (Figure 1).

Table 4 illustrates physicians’ practices in antibiotics deprescribing. Notably, 46.9% of respondents (n = 117) reported regular assessment of the need for deprescribing, while 42.9% (n = 108) assessed antibiotics for potential patient risks. Educating patients about potential antibiotic-related harm was reported by 40.5% of physicians (n = 102). Recommendations for antibiotic deprescribing, especially when patient preferred not to continue antibiotics, were always made by 29.4% (n = 74). A preference for delayed antibiotic prescribing over deprescribing when antibiotics were considered unnecessary was indicated by 34.5% of the participants (n = 87). Additionally, 28.2% (n = 71) would only deprescribe if patient had taken antibiotics for at least five days, and 29% (n = 73) agreed to deprescribe to reduce antimicrobial resistance. Furthermore, 23.4% (n = 59) agreed to withdraw antibiotics initiated by patients themselves, and 31.3% (n = 33) agreed to do so if antibiotics were obtained without prescription from a pharmacy.

**Table 4.** Physicians’ practice in antibiotic deprescribing (n = 252).

Statement	Always n (%)	Often n (%)	Sometimes n (%)	Rarely n (%)	Never n (%)
I regularly assess the need for antibiotic deprescribing in my patients.	117 (46.9)	98 (38.9)	34 (13.5)	2 (0.8)	1 (0.4)
I assess antibiotics for potential risk for patients.	108 (42.9)	107 (42.5)	30 (11.6)	5 (2.0)	2 (0.8)
I educate patients regarding the risk of harm related to potentially inappropriate antibiotics.	102 (40.5)	97 (38.5)	45 (17.9)	7 (2.8)	1 (0.4)
I recommend antibiotic deprescribing, especially when the patient wishes not to take the antibiotic course.	74 (29.4)	110 (43.7)	48 (19.0)	13 (5.2)	7 (2.8)
I prefer using delayed antibiotic prescribing rather than deprescribing an antibiotic when I consider the antibiotic is not needed.	87 (34.5)	99 (39.3)	49 (19.4)	10 (4.0)	7 (2.8)
I would only deprescribe antibiotic if the patient has already taken it for at least five days.	71 (28.2)	116 (46.0)	42 (16.7)	13 (5.2)	10 (4.0)
I agree to deprescribe if this shows to reduce antimicrobial resistance.	73 (29.0)	121 (48.0)	47 (18.7)	7 (2.8)	4 (1.6)
I would act differently regarding antibiotic deprescribing during a phone consultation compared to a face-to-face visit.	67 (26.6)	119 (47.2)	54 (21.4)	8 (3.2)	4 (1.6)
I would agree to withdraw an antibiotic regimen if it was initiated by the patient	59 (23.4)	140 (55.6)	40 (15.9)	8 (3.2)	5 (2.0)
I would agree to withdraw an antibiotic regimen if it was given at the pharmacy without a prescription	79 (31.3)	108 (42.9)	47 (18.7)	9 (3.6)	9 (3.6)
Inability to obtain pharmacist input on antibiotic deprescribing	33 (13.1)	37 (29.0)	91 (36.1)	49 (19.4)	6 (2.4)

Logistic regression analysis for factors influencing associated with physicians’ willingness to deprescribe antibiotics (Table 5) showed that none of the studied demographic factors had a significant association with physicians’ willingness to deprescribe antibiotics (*p* > 0.05 for all).

**Table 5.** Assessment of factors associated with physicians’ willingness to deprescribe antibiotics (n = 252).

Parameter	Willingness to deprescribe antibiotics [0: Rejecter/Hesitant, 1: Acceptor]			
	COR	*P*-value#	AOR	*P*-value$
Age (years)	0.251	0.028*	0.978	0.272
Gender				
Females	Reference	0.468	–	–
Males	0.710			
Title				
General Practitioner	Reference	0.562	–	–
Junior Resident (1st or 2nd year of residency)	1.342	0.841	–	–
Senior Resident (3rd or 4th year of residency)	1.107	0.017	–	–
Consultant	0.337			
Years of experience	0.235	<0.001*	1.079	0.082
Country of graduation				
Jordan	Reference	0.089*	0.749	0.439
Others	0.448			
Number of daily consultation				
<10	Reference	0.396	0.792	0.602
10–20	0.699	0.075*	0.482	0.118
>20	0.469			

Notes: COR: Crude odds ratio, AOR: adjusted odds ratio. # using simple logistic regression, $ using multiple logistic regression, ^ eligible for entry in multiple logistic regression, * significant at 0.05 significance level.

## 4. Discussion

Although deprescribing is mainly linked to chronic medical conditions, the minimisation of unnecessary antibiotic use is equally important. Antibiotic deprescribing reduces antimicrobial resistance and reduces the risk of adverse events of antibiotics that have been prescribed for sufficient appropriate duration or not indicated (Hansen et al., 2019). Since physicians assume the responsibility of deprescribing, it is imperative to explore their knowledge of and attitudes toward the deprescribing process. Physicians in our study were aware of the importance of antibiotic deprescribing and its influence on the emergence and spread of resistance. Similar findings were revealed in studies conducted in Nigeria and Saudi Arabia (Akande-Sholabi et al., 2023; AlRasheed et al., 2018). However, evidence from a randomised controlled trial showed that the clinical outcomes of patients who underwent antibiotic deprescribing were similar to those who continued the antibiotic therapy (Llor, Moragas, et al., 2022).

The majority of physicians in this study were unwilling to deprescribe antibiotics, they were concerned that patients may not be receptive and may not welcome deprescribing. In consistence, some physicians believed that discontinuation of antibiotics might harm their relationship with their patients (Urbiztondo et al., 2018). Furthermore, patients’ negative perception was reported as a deprescribing barrier owing to their self-interpretations of this intervention as a demission of patient care and a process for cost savings (Okeowo et al., 2023). On the other hand, Lukacena et al revealed that 83.5% of their study population expressed willingness to deprescribing if it was approved by their clinicians (Lukacena et al., 2022). Physicians also expressed their concerns that antibiotic deprescribing may increase complications and hospital admissions. However, in general, hospital admissions and increased length of stay are caused by adverse drug reactions caused by polypharmacy which in turn can be reduced by deprescribing (Formica et al., 2018). Moreover, in this study, more than two-thirds of the physicians believed that deprescribing should be included in clinical guidelines. This perception was similarly reported by health professionals in other studies (Reeve et al., 2017; Wu et al., 2021). Han Kua et al. (2019) revealed that health professional considered systemic practices of deprescribing and educational tools as enablers of the process. Health professionals require guidelines and explicit algorithms that provide sufficient guidance and evident steps in the process that guarantee patient safety and satisfaction. Indeed, the presence of specific guidelines would represent a reference point for all parties involved in the management of the patient care process.

Many structured deprescribing guidelines are available but are not confined to a certain drug and none were assessed by randomised controlled trials to provide evidence that these measures have positive effects on the clinical outcomes (Scott et al., 2017). Many guidelines which are in the form of recommendations, and communication between the healthcare professionals, caregivers, and patients are highly encouraged to manage the care plan and reduce emergency admissions (Crisafulli et al., 2022). Guidelines for deprescribing implementation in older patients’ setting are abundant (O'Mahony et al., 2015; Thompson et al., 2019), mostly were developed to overcome the problem of polypharmacy which is very common encountered in this population and resulted in higher rate of visits to the emergency department (Doumat et al., 2023). Moreover, antibiotics are overprescribed for many asymptomatic elderly patients suspected of urinary tract infection (Álvarez Artero et al., 2019). Consequently, deprescribing is one possible strategy to reduce polypharmacy and improve safety and mortality, if applied in the form of patient-specific interventions (Page et al., 2016).

Barriers to deprescribing may be related to the patient, physician, and/or the medical institution (Gupta et al., 2019). Physicians in this study reported insufficient time as a challenge in the deprescribing process since these comprehensive conversations with the patients are time consuming and may not be feasible in some clinical settings.

Lack of communication and inability to gain information from other relevant medical resources is another barrier perceived by the participants. Deprescribing is a multifaceted process and requires the cooperation of various medical specialties, which necessitates better communication at the organisational and personal level (Pereira & Veríssimo, 2023). The absence of a full profile of the patient medical condition can pose a potentially significant impediment to deprescribing (Akande-Sholabi et al., 2023). A research project in the United Arab Emirates assessed the knowledge, attitudes, and practices of community pharmacists regarding deprescribing, identifying numerous obstacles they encounter, including significant resistance from patients and insufficient resources (El-Dahiyat et al., 2023). This highlights the necessity for training workshops and enhanced interdisciplinary collaboration to enhance deprescribing methods to guarantee medication safety (El-Dahiyat et al., 2023; Nashwan et al., 2024).

Physicians believed that patients may not welcome a de-escalation in their medication. However, Gilpin et al. (2022) revealed that hospital inpatients had positive attitudes toward deprescribing and did not interpret this process as abandoning them. To achieve that, the shared decision to deprescribe must involve the patients and their caregivers after informing them of potential benefits (Jansen et al., 2016). Unfortunately, this shared decision-making is not an easy process and engaging patients, especially the elderly, can be difficult and needs training, communication skills, and guidance which is not available (Bynum et al., 2014).

Most physicians reported that withdrawing medications prescribed by another clinician is an ethical challenge. This was also considered as a potential ethical barrier to deprescribing by Norton et al. (2023) in 3000 primary care physicians who care for older adults with dementia. Physicians are more eager to deprescribe antibiotics initiated by themselves than when the therapy was initiated by others (Llor, Cordoba, et al., 2022). It is considered a ‘professional etiquette’ not to controvert the decision of another colleague and considered culturally unacceptable (Doherty et al., 2020). The participants in our study were young and mainly general practitioners, specialised and senior clinicians may be more confident in reassessing the decision of other colleagues and stopping unnecessary antibiotics.

Strengths of the study include the fact that this is the first study in Jordan to evaluate the knowledge and attitudes of physicians towards deprescribing since they are the main prescribers. The study provided an understanding of the challenges that face deprescribing process which must be addressed for successful implementation. Limitations include the relatively young age of the participating physicians, and more than half of participants were general practitioners which confines the representation of older, more experienced, and specialised physicians. The use of electronic forms through social media did not allow us to obtain a response rate. Another limitation is that attitudes are different than actual practice, despite the positive attitude we cannot anticipate similar behaviour in the real world.

Future work should assess the actual practice of deprescribing antibiotics, the guidelines used, and the clinical outcomes of this process. Additionally, assessing the predictors of antibiotic deprescribing practice whether related to the physician, the patient, or organisation can provide important information.

## 5. Conclusion

Physicians in Jordan are aware of the deprescribing process of antibiotics and recognise its benefits. Additionally, many received training in antibiotics deprescribing which provides a strong basis for the implementation of this process. However, deprescribing is a transverse process that requires the involvement of all healthcare professionals to insure a clear overall clinical picture of the patient and a rigorous follow up. Effective communication with the patients and their caregivers is also imperative to engage them in the process to achieve shared decisions. The challenges on the patient, physician, and organisational levels that must be addressed and requires the establishment of policies that guarantee the implementation of tools and guidelines for deprescribing.

## Data Availability

The data that support the findings of this study are available from the corresponding author upon reasonable request.
